# Angiogenesis and Re-endothelialization in decellularized scaffolds: Recent advances and current challenges in tissue engineering

**DOI:** 10.3389/fbioe.2023.1103727

**Published:** 2023-02-16

**Authors:** Radman Mazloomnejad, Amirhesam Babajani, Mohammadreza Kasravi, Armin Ahmadi, Siavash Shariatzadeh, Soheyl Bahrami, Hassan Niknejad

**Affiliations:** ^1^ Department of Pharmacology, School of Medicine, Shahid Beheshti University of Medical Sciences, Tehran, Iran; ^2^ Department of Surgery, University of California Los Angeles, Los Angeles, CA, United States; ^3^ Ludwig Boltzmann Institute for Experimental and Clinical Traumatology in AUVA Research Center, Vienna, Austria

**Keywords:** decellularization, endothelialization, angiogenesis, scaffold, stem cells, translational medicine

## Abstract

Decellularization of tissues and organs has recently become a promising approach in tissue engineering and regenerative medicine to circumvent the challenges of organ donation and complications of transplantations. However, one main obstacle to reaching this goal is acellular vasculature angiogenesis and endothelialization. Achieving an intact and functional vascular structure as a vital pathway for supplying oxygen and nutrients remains the decisive challenge in the decellularization/re-endothelialization procedure. In order to better understand and overcome this issue, complete and appropriate knowledge of endothelialization and its determining variables is required. Decellularization methods and their effectiveness, biological and mechanical characteristics of acellular scaffolds, artificial and biological bioreactors, and their possible applications, extracellular matrix surface modification, and different types of utilized cells are factors affecting endothelialization consequences. This review focuses on the characteristics of endothelialization and how to optimize them, as well as discussing recent developments in the process of re-endothelialization.

## 1 Introduction

Increased organ failure due to chronic infections, diseases, and genetic problems has elevated organ transplant requirements in recent years. According to the Global Observatory of Donation and Transplantation, approximately 150 thousand organs were transplanted worldwide in 2019 (Graham et al., 2022). Also, the United States organ donation statistics revealed that more than 106,000 patients were awaiting lifesaving organ transplants, and another person was added to the nation’s organ transplant waiting list every 9 min (Organ donation, 2023). Organ donation contraindications, long waiting lists, and mismatched donor and recipient statistics are challenging organ transplantation issues. Besides, a lack of existing therapies and limitations associated with transplantation procedures (e.g., post-surgery isolation and long-term immunosuppression therapy) may predispose patients to severe infections, malignancies, and transplant rejection (Halloran et al., 2022). Thus, these challenges call for researchers to suggest alternatives to conventional transplanting methods.

Tissue engineering and regenerative medicine, aiming to design, produce, and achieve an efficient and functional organ, play a crucial role in overcoming immunity limitations and transplantation obstacles (Ahmadi et al., 2022; Li et al., 2022a; Cox-Pridmore et al., 2022). Decellularization and recellularization (D&R) of tissues and organs are efficient alternatives to classic organ transplantation (Dias et al., 2022). The purpose of decellularization is to wash the complete cell content of the target organ or tissue to achieve a cell-free extracellular matrix (ECM) scaffold while preserving parenchymal and vascular structures within this scaffold (Shahraki et al., 2021; Uday Chandrika et al., 2021). Besides, D&R are performed to attain functional tissues and organs and prevent an immune response against the designed structure and its components. Despite the studies and advances in this field, there are still many limitations in generating a wholly functional and viable human organ or tissue by utilizing D&R.

One of the critical limitations of the D&R methods is undesired organ nutrition, blood supply, and lack of functional vessels, which may make the graft unsuitable for transplantation and further hamper achieving functional organs (Li et al., 2022a). Functional blood vessels can be defined as lumens within the ECM, covered by a monolayer of endothelial cells (ECs), providing a non-thrombogenic surface in the circulation system (Naito et al., 2020). Lack of a consistent endothelium layer leads to exposure of ECM components to the coagulation system after transplantation, providing a condition for direct platelet-collagen interaction (Li et al., 2022a). Consequent platelet aggregation leads to clot formation and impedes blood flow to the organ. In turn, blood flow restriction causes cell hypoxia and cell death, which damages the organ functionality (Xie et al., 2021; Nazerian et al., 2022). In order to preserve blood flow within the organ without any clot formation and to optimize the engineered organ’s viability, the acellular vasculature must be completely re-endothelialized (Li et al., 2022a).

Multiple strategies and processes have been adopted for this goal and to respond to the challenges described above, each of which separately plays a vital role in the endothelialization process (Seiffert et al., 2021; Daryabari et al., 2022; Haeublein et al., 2022; Kim et al., 2022). Decellularization and its protocols are among the first influential factors in the endothelialization of acellular structures. They exert their effect due to the degree of success in removing cellular materials, as well as their impact on the acellular scaffold and ECM microstructure alterations. So that the more intact the obtained acellular structure is, the better the endothelialization outcome will be obtained (Daryabari et al., 2022). This point is also valid in recellularization conditions. In this respect, bioreactors and the cellularization procedures, which are used to simulate the native niche as much as possible regarding better endothelialization, are other essential factors in the endothelialization of acellular scaffolds (Gorman et al., 2018). Additionally, surface biofunctionalization (Dal Sasso et al., 2021; Keshi et al., 2021) and utilized cells play a crucial role in regulating the cells’ behavior and ultimately increasing the ECs coverage of the acellular vasculature.

**FIGURE 1:**
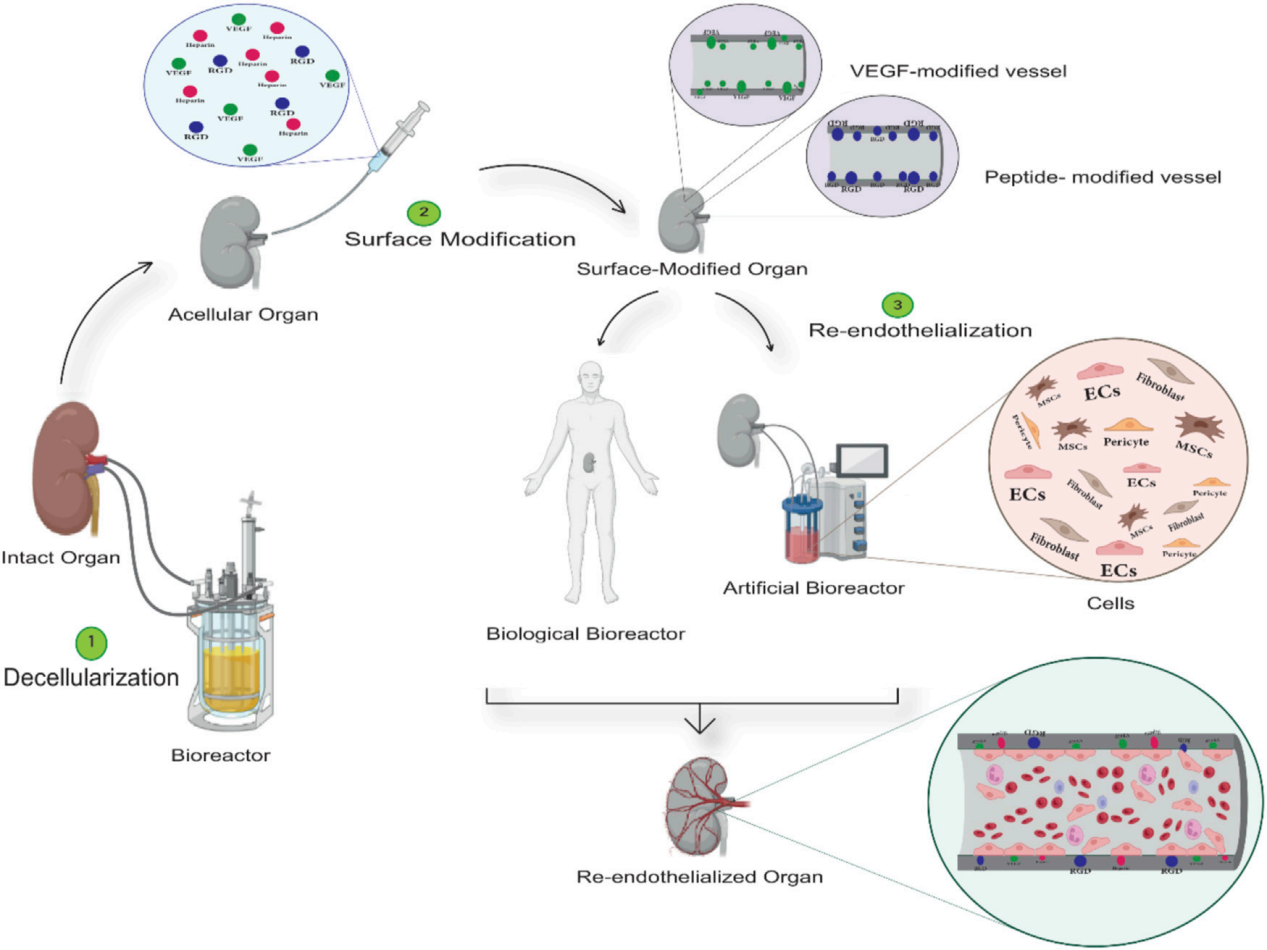
General structure of tissue decellularization and re-endothelialization procedures. In the first stage (Step 1), the decellularizing agents, such as detergents, are injected into the target tissue using bioreactors. The acellular structure is obtained by controlling settings such as material concentration, time and temperature, etc. In the second stage (Step 2), the acellular structure obtained gets modified by bioactive molecules to minimize the damages caused by the decellularization process and improve the vascular structure’s endothelialization outcome. Ultimately, in the process of endothelialization (step 3), artificial and biological bioreactors are employed to use generated flow and cell cocktails, respectively, or by transplanting the structure to a living organism and using its blood flow and progenitor cells to cover the intended vascular structure with the endothelial cell.

Ultimately, on the track of acellular scaffold recellularization and endothelialization, the identification of the optimal decellularization method along with scaffold features, the bioreactor utilization approach, cytokine, and growth factors (GFs) quantities, appropriate cell type, and cell density are the most critical determinants of organ engineering (Chan et al., 2022). In this review, we intend to discuss the most recently utilized approaches focusing on the roles of optimized decellularization, bioreactors, surface modification, and cells on angiogenesis and endothelialization of acellular vascular structures (which are briefly shown in Figure 1.).

## 2 The role of optimized decellularization in favorable endothelialization

The endothelialization outcome depends on preserving ECM microstructure and component composition during decellularization. Thus, the more intact the ECM remains, the more desirable tissue engineering outcomes are achieved (Daryabari et al., 2019).

As the end product of decellularization, ECM is a non-living, non-cellular biomaterial that supports the resident cells as a particular, functional, and protective substrate (Rijal, 2017; García-Gareta et al., 2020; Sani et al., 2022). This three-dimensional stroma provides structural and elastic properties, induces necessary biochemical, biophysical, and biomechanical signals for tissue morphogenesis, differentiation, and homeostasis, as well as establishes an appropriate environment for the distribution and transport of nutrients and oxygen to the cells (Hussey et al., 2018; García-Gareta et al., 2020; Sani et al., 2022). The two main components of the vascular ECM are the basement membrane (BM) and the interstitial ECM. The basement membrane (BM) predominantly possesses elements such as collagen IV, fibronectin, laminin, heparan sulfate, and proteoglycans, and the interstitial ECM contain mainly elastic fibers and types of collagens, including collagen type I. Both BM and interstitial ECM have been studied in endothelialization (Senk and Djonov, 2021; Yu et al., 2021). It has been found that BM components, which act as an anchor for cells, play a key role in ECs attachment, migration, proliferation, and angiogenesis (Senk and Djonov, 2021). In addition to the preservation of ECM, complete cell removal is beneficial in acquiring optimal decellularized scaffolds (Maghsoudlou et al., 2013). *In this regard, decellularization on a measurable scale is determined as: 1. less than 50 ng of dsDNA per mg of ECM (dry weight), 2. less than 200 bp retained DNA fragment length in acellular ECM, and 3. the absence of visible nuclear material under hematoxylin and eosin (H&E) and 4′,6-diamidino-2-phenylindole (DAPI) staining* (Crapo et al., 2011).

The obtained ECM-preserved acellular scaffold with intact microarchitecture and significant reduction of immunogenic antigens is remarkably useful for tissue engineering in various aspects, such as inducing parenchymal cell growth and angiogenesis (Uygun et al., 2010; Gholipourmalekabadi et al., 2015; Peloso et al., 2015). Many studies have proven the supporting role of the acellular matrices obtained from the decellularization process in angiogenesis and endothelialization (Maghsoudlou et al., 2013; Mei et al., 2016; Katsimpoulas et al., 2019). For instance, an acellular rat scaffold provides a regenerative microenvironment that supports human umbilical vein endothelial cells (HUVECs) proliferation and attachment (Mei et al., 2016). Another study indicated that decellularized ovine arteries increased mesenchymal stem cell (MSCs) proliferation and induced endothelial differentiation (Zhang et al., 2016). Moreover, some evidence supports the positive effects of decellularized scaffolds in *in vivo* angiogenesis (Alvarèz Fallas et al., 2018).

**FIGURE 2:**
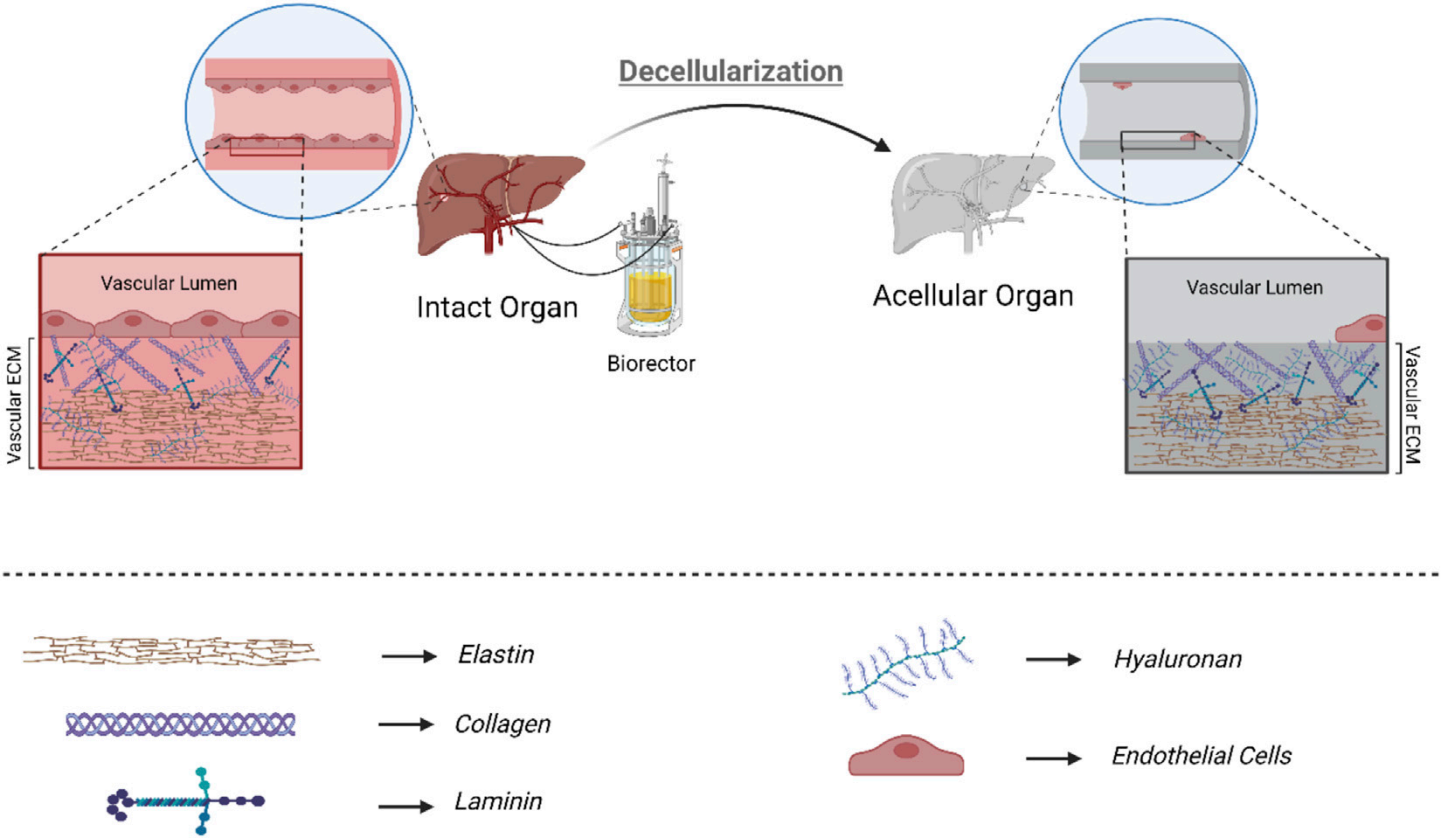
Detergent-based decellularization: Optimized detergent-based decellularization of biological scaffolds is done to clear away cells and cell debris completely, removing potential immunogens and antigens present in the scaffold while preserving the structural and functional proteins that make up the extracellular matrix. Considering the ECM obtained from decellularization and the importance of the components present in ECM in regeneration, the more intact the final product of decellularization is, the better the results of recellularization, endothelialization, and final tissue function will be.

Furthermore, studies have shown that endothelialization outcomes vary depending on the chosen decellularization technique (Maghsoudlou et al., 2013). Regarding the angiogenesis, detergent-based decellularization techniques have been extensively used (Figure 2). The type of detergents in the decellularization process and their biochemical properties also impact ECM integrity and the endothelialization outcome (Schmitt et al., 2017). Detergents with uncharged hydrophilic headgroups (non-ionic detergents) in decellularization are less able to break protein-protein bonds; therefore, they are mainly used to break lipid-lipid and lipid-protein bonds. Triton X-100 is the most utilized non-ionic detergent for disrupting lipid-lipid and lipid-protein interactions without affecting protein-protein bonds. Therefore, utilizing non-ionic detergent results in less damage to the ECM, which contains a considerable protein content and protein-protein bonds (Crapo et al., 2011). Studies have also shown that Triton X-100, a non-ionic detergent, could successfully decellularize various organs such as the liver, kidney, lung, spleen, and pancreas (Zhou et al., 2016a; Ghiringhelli et al., 2018; Baptista et al., 2011; Dall’Olmo et al., 2014; Wu et al., 2016; Nagao et al., 2013; Dew et al., 2016). Triton X-100 for liver decellularization resulted in an acellular hepatic scaffolding with 97% DNA elimination and a maintained microstructure (intact, patent, and fine-branching vascular network). After perfusing endothelial cells within the acellular scaffold, the results represented a significant advancement in endothelialization and whole-organ bioengineering (Baptista et al., 2011). Detergents with charged hydrophilic headgroups (Ionic agents) are remarkably effective during decellularization and DNA/cytoplasmic membrane solubilization (Weymann et al., 2013; Peloso et al., 2015; Hussein et al., 2016; Mao, 2017; Schmitt et al., 2017; Koenig et al., 2019). Compared to Triton X-100, SDS, an ionic detergent, is more capable of removing the pathogenic and antigenic epitopes, leading to more complete decellularization (Hussein et al., 2016). Several groups have successfully utilized SDS to achieve acellular whole organ scaffolds. Mao et al. also decellularized the porcine liver using SDS perfusion decellularization and successfully endothelialized the acellular scaffold by perfusing human and porcine umbilical vein endothelial cells under controlled conditions (Mao, 2017).

The other affecting parameter in detergent-based decellularization is the route of detergent utilization and their delivery system. There are different methods for delivering decellularization agents, including immersion agitation and perfusion (Yang et al., 2022). Many studies have utilized bioreactors and perfusion to infuse detergent solutions through organ ducts, such as organ vasculature (Zhang et al., 2015). Perfusing a detergent solution through the organ vascular network leads to a broader cell-detergent contact rather than agitation and diffusion alone (Baptista et al., 2011; Ko et al., 2014). Also, the perfusion decellularization methods have been found effective for cell and residual DNA removal while preserving the microarchitectural integrity of the native organ ECM (Ko et al., 2014; Mao, 2017). It has been shown that the perfusion decellularization protocol could be applied to rats, mice, and pigs’ hearts to achieve an acellular scaffold with maintained microarchitecture and patent vasculature (Robertson et al., 2014). In this regard, the detergent solution was perfused through the liver vascular network to decellularize the liver with much better detergent distribution through the entire liver (Baptista et al., 2011). This gentle procedure led to decellularizing ECM beside the preserved architecture of the liver matrix and vascular system. The organ vascular network was kept intact, patent, and capable of supporting the entire organ’s perfusion (Stabler et al., 2016).

In addition to detergent-based decellularization, other chemical agents such as acids and bases, chelating substrates and enzymes, are also employed to remove cells from organ scaffolds (Gilbert et al., 2006). Peracetic acid (PAA) and sodium hydroxide (NaOH), two widely used chemical agents (Wang et al., 2015; Poornejad et al., 2016), as well as enzymes such as nucleases, trypsin, collagenase, lipase, dispase, thermolysin, and α-galactosidase are examples of agents used in decellularization processes (Crapo et al., 2011; Gilpin and Yang, 2017). These agents can help in the removal of immunogenic cell materials, resetting the ECM to a neutral pH, and enhancing the efficacy of the decellularization process. However, their use can also lead to damage to the ECM, reducing the content of fibronectin, laminin, elastin, and GAGs, and ultimately, impacting the integrity of the scaffold, which can interfere with post-decellularization re-endothelialization (Schenke-Layland et al., 2003; Tsuchiya et al., 2014; Poornejad et al., 2016). Moreover, physical methods such as sonication have been proposed to enhance the tissue’s exposure to decellularization reagents (Lin et al., 2021; Nayakawde et al., 2021). Recent studies have employed ultrasound in detergent-based decellularization to divide cellular components from the ECM, diminishing the need for higher detergent concentrations. However, prolonged use of high-energy ultrasonic treatment can be detrimental, leading to tissue fragility, weakness, and leakage (Lin et al., 2021). Although these methods have been employed in decellularizing many organs and tissues, there is a lack of sufficient data about their usage and effects on endothelialization. Hence, given the limited data available showing the impact of these approaches on ECM structure, its components, and vascular endothelialization, further research is necessary to address these limitations.

Considering the effectiveness of perfusion decellularization in cell washing, there is a requirement for establishing a perfusion platform to provide proper solution flow within the organ ducts. Studies have revealed the unique role of bioreactors in establishing the flow of detergent solutions and regenerative cocktails in the field of decellularization and endothelialization, respectively.

## 3 The role of bioreactors in favorable endothelialization

Homogenous cell settlement and appropriate cellular proliferation, migration, and differentiation are critical for better endothelialization and complete cell coverage of vasculature (Ko et al., 2014). Furthermore, a proper culture environment after cell seeding is necessary to improve the results of endothelialization. The post cell-seeding culture can be implemented in a static manner or *via* the perfusion method, defined as delivering fresh media and removing waste products. Perfusion re-endothelialization has highlighted the attachment ability of ECs to the luminal surface of blood vessels and facilitated the spreading of initially adhered cells (Ko et al., 2014; Mao, 2017). On the other hand, static culture does not benefit from this flow because cultivation is done in a fixed environment without the perfusion and circulation of the medium.

The bioreactors attempt to simulate a physiologic microenvironment by creating a controllable flow rate, solution pH, oxygen pressure, temperature, and pressure. Several studies have verified the efficiency of perfusion re-endothelialization and bioreactors compared to the static method (Yazdani et al., 2010; Xie et al., 2012; Matsugaki et al., 2013). A study compared static, and perfusion cultures to re-endothelialize decellularized lung scaffolds (Scarritt et al., 2018). Although static culture led to ECs attachment to the rat lung vasculature, these cells did not spread well within the vascular network. Besides, cell proliferation and survival rates were lower than in perfusion culture, and the endothelial cells were non-uniformly distributed. In addition, cell aggregation blocked many vessels during static culture. Conversely, in the perfusion culture method with a peristaltic pump, exposure to low levels of fluid flow, helped better cells spread within vessels with little cell aggregation and lumen clogging (Scarritt et al., 2018).

The bioreactors used in the re-endothelialization studies can be divided into (Graham et al., 2022) artificial and (Organ donation, 2023) biological bioreactors. Artificial bioreactors are built-in bioreactors used in laboratory environments and *in vitro* studies. Biological bioreactors are also defined as utilizing an *in vivo* host circulation system to obtain appropriate recellularization.

### 3.1 The role of artificial bioreactors in endothelialization

Recent studies have used artificial bioreactors to recellularize acellular organs or tissues and fabricate relatively functional engineered tissues. These perfusion-dependent bioreactors can bio-mimic the biological, chemical, or mechanical aspects of the intended organ microenvironment by providing a platform with controlled settings such as oxygen tension, temperature, pH, and shear stress (Martin et al., 2004; Nagel-Heyer et al., 2005). Therefore, considering the characteristics of intended organs and adapting the settings of artificial bioreactors are essential to achieve the optimum tissue engineering results (Ratcliffe and Niklason, 2002). Some factors, such as oxygen tension, perfusion protocol, and the chosen perfusion route, should be modified and regulated to achieve better-endothelialized grafts (Moeinabadi-Bidgoli et al., 2021). In the case of oxygen tension, it has been observed that culturing human adipose-derived stromal cells (ASCs) on decellularized adipose tissue (DAT) scaffolds in a perfusion bioreactor under hypoxia increased cell growth and significantly enhanced angiogenesis in the acellular structure (Han et al., 2021). The flow rate is the other controllable factor that plays a crucial role in artificial bioreactor application to optimize engineered tissue endothelialization. Three perfusion methods used in bioreactor applications during the recellularization process are static perfusion (a constant flow rate), ramping perfusion (increasing and decreasing flow rates), and combining static and ramping perfusion cell seeding (Ko et al., 2012).

The initial approach to endothelialize decellularized vascular structures is static flow recellularization. Studies have attempted to endothelialize acellular vasculature by exposing them to a low flow rate of cell cocktail (1–3 ml/min) (Song et al., 2013; Scarritt et al., 2018). Hence, Scarritt ME et al. have looked into and compared the use of steady flow rates in re-endothelialization. They represented that using a flow rate of 3 ml/min in the endothelialization of the acellular rat lung’s vascular structures leads to more cell apoptosis and a decrease in cellularization compared to 1 ml/min flow (Scarritt et al., 2018). In addition, a 3 ml/min flow rate yields an increase in pressure in the vascular structures and, as a result of fragmentation of the cells, damage, and rupture of the vascular structures, followed by cell leakage into the parenchyma. On the other hand, although in employing a flow of 1 ml/min, the apoptosis of cells was less, due to the lower pressure in the blood vessels, it was not possible to access and endothelialize the end parts of the organ (Scarritt et al., 2018). Therefore, according to the limitations of applying a static flow rate in the endothelialization of acellular structures, ramping perfusion methods have been used in order to achieve complete endothelialization.

One of the significant advantages of utilizing ramping perfusion is flow fluctuation that opens up collapsed capillaries and re-endothelializes these unreachable vessels to promote accessibility and recellularization (Lee et al., 2009; Le et al., 2017). The investigations revealed that gradually raising the perfusion rate for the re-endothelialization promotes ECs survival and improves the cell spreading outcomes (Stabler et al., 2016). Additionally, a moderate increase in perfusion flow rate can promote cellular alignment and settlement, keep the vascular network (even microvessel lumens) open, and prevent the aggregation of injected cells within the acellular vasculature. Thus, many studies have utilized ramping perfusion to endothelialize the acellular vessels of various organs. Various numerical ranges and policies have been employed in the ramping perfusion application. Lower levels of flow rates (1–10 ml/min) are most commonly utilized in endothelializing various decellularized organs, including the liver, heart, lung, and kidney (Petersen et al., 2010; Robertson et al., 2014; Devalliere et al., 2018; Meng et al., 2019). For instance, successful endothelial coverage was achieved by perfusion of ECs through the pulmonary vessels of decellularized rat lungs at a rate of 1–5 ml/min (Petersen et al., 2010). Similarly, considering the endothelialization of acellular rat liver, applying a flow rate of 2 ml/min and then increasing it to 8 ml/min yielded proper endothelialization (Devalliere et al., 2018).

Nonetheless, a broader numerical range has also been employed for endothelialization. For instance (Ko et al., 2014), In Kap Ko et al. perfused the cell suspension into the renal artery at a flow rate of 2 ml/min and gradually increased it to 20 ml/min to reduce cell accumulation and overcome vascular occlusion (Ko et al., 2014). The researchers chose 20 ml/min as the maximum utilized flow rate because recent studies have shown that flow rates higher than 20 ml/min did not improve the endothelialization of the vasculature and prevented cells from attaching to the vascular network ECM (Ko et al., 2014). It can be concluded that a high or rapidly increased fluid flow rate not only fails to improve the endothelialization outcome like a gradual increase but also may lead to cell detachment, cell death, and reduced coverage (Ko et al., 2014).

In addition to flow rate, it has been revealed that a gradual increase in shear stress in the endothelialization process can improve ECs retention and create a complete endothelial-like monolayer both *in vitro* and *in vivo*. Further, it can improve cell survival and strengthen cell-cell and cell-ECM connections (Jiao et al., 2022). Thus, several studies have tried to explore shear stress and pressure characteristics to improve cell conditions and endothelialization outcomes. Yazdani et al. suggested that cyclic preconditioning with oscillating pressure, flow, and mechanical strain is a highly effective method for inducing cell proliferation and alignment of multilayer vascular smooth muscle cells (VSMCs). It seems that the increase in expression and secretion of GFs and signaling molecules from VSMCs and their proliferation and alignment enhancement result from oscillating preconditioning and mechanical stress (Yazdani et al., 2009). Regarding the cell’s functionality, upregulation in the protein expression of endothelial nitric oxide synthase and prostaglandin I synthase indicates the function of ECs. Also, an elevated ECs resistance to blood flow-induced shear stress was observed in the cyclic preconditioned scaffold compared to the steady shear stress (Yazdani et al., 2010).

Although the efficacy of bioreactor variables such as flow rate, pressure, and shear stress on the phenotypic characteristics of ECs and endothelialization has been explored, the optimal conditions to achieve an integrated vascular structure in acellular scaffolds have yet to be determined. Consequently, further studies examining these factors are necessary to determine the appropriate range for each in the endothelialization process and normalize them to the tissue mass of various organs.

Another topic that impresses recellularization is the route by which cells are infused and the direction of infusion (Dew et al., 2016). As the decellularization process is done by infusing the detergent solutions throughout the scaffold’s vascular network and the wide contact surface obtained through the vessels, recellularization should be done through the vasculature, which leads to much more completely recellularized scaffolds (Dew et al., 2016). It has been observed HUVECs perfused through the pulmonary vessels of cadaveric lungs resulted in ECs deposition and endothelialization of the acellular vasculature (Ott et al., 2010). Considering the direction of blood flow inside the vessels, cells are injected through them in two approaches: antegrade (same direction) and retrograde (opposite direction).

Given the importance of having a complete perfusion circuitry in the re-endothelialization of vascular trees within decellularized matrices, mainly when using a bioreactor setup, studies have revealed that arterially implanted cells are spread diffusely throughout the scaffold to the distal parts, while cells injected through veins tend to be mainly located in the proximal regions (Scarritt et al., 2018). Thus, many investigations have only injected cells into acellular scaffolds *via* the arterial route (Bonandrini et al., 2014; Du et al., 2016; Xue et al., 2018). Other studies have tried investigating endothelialization by implanting ECs from both arterial and venous routes. As an example, to recellularize acellular rat heart vasculature, rat aortic endothelial cells have been infused in three directions: (Graham et al., 2022): retrograde aortic infusion, (Organ donation, 2023), antegrade brachiocephalic artery (BA) infusion, and (Halloran et al., 2022) retrograde inferior vena cava (IVC) and antegrade BA combined infusion (Robertson et al., 2014). It was observed that re-endothelialization improved with cell delivery through combined IVC + BA routes than single-route retrograde aortic or antegrade BA infusion. Combined IVC + BA infusion increased scaffold and vessel cellularity within the decellularized heart scaffold. Also, in another study, Ren et al. observed a similar outcome in an attempt to re-endothelialize the rat pulmonary vasculature. They reported better cell coverage and much more ECs distribution along the pulmonary vasculature when the ECs perfused through the pulmonary artery and then the veins (Ren et al., 2015). Thus, it can be concluded that utilizing both venous and artery infusion leads to a more homogenous cell distribution and much better cell coverage in the re-endothelialization of the whole organ (Robertson et al., 2014).

Moreover, it must be acknowledged that the methods of decellularization used can cause significant damage to the structure of the decellularized vascular tissue (Stabler et al., 2016; White et al., 2017). As a result, the perfused tissue will have a leaky structure in the absence of an efficient venous and lymphatic system (Ren et al., 2015). Studies of the continuity and integrity of the vascular bed in acellular rat lungs and kidneys have revealed a degree of vascular permeability, media leakage, and a reduction in hydrostatic pressure along the vessels (Stabler et al., 2016; Haeublein et al., 2022). This leads to media leakage into the parenchyma, reducing the efficiency of cell delivery and diminishing the endothelialization rate (Stabler et al., 2016). Therefore, future studies should focus on optimizing the conditions of recellularization, reducing the leakage rate of structures, and effectively collecting and handling leaked culture media while maintaining sterility and aeration conditions.

### 3.2 The role of biological bioreactors in endothelialization

A biological bioreactor refers to a living system that supports and facilitates the growth and development of cells or tissues. In the field of acellular scaffold recellularization, the host body is considered a biological bioreactor due to its circulation system and internal environment, which have the capability to support cell regeneration, differentiation, and rearrangement within acellular scaffolds (Khorramirouz et al., 2017). Studies have shown that the physiological capacities of the body can coordinate and improve cell survival and behavior by providing a stable and protective environment for tissues and organs. This makes the body an ideal platform for *in situ* vascularization (Hashemi et al., 2018). This approach relies on the surgical expertise needed to implant the engineered tissue or organ within a fully vascularized and supportive pocket (like an omentum or muscle flap) within the body for an extended period to be connected to the native circulation system and become completely vascularized (Khorramirouz et al., 2017; Hashemi et al., 2018). The presence of cytokines and GFs, along with neovasculature and nerve induction within the implanted engineered tissue, are some advantages of this method that make it a promising and practical procedure (Khorramirouz et al., 2017).

Additionally, *in vivo* tracheal tissue engineering study showed that animals’ bodies could be utilized as potential bioreactors when transplants are in a normal anatomical position (Zhang et al., 2015). In a recent study, researchers transplanted an acellular kidney and ureter to a rat model to re-endothelialize and generated vessels using the body as a biological bioreactor. Many vessels were endothelialized, and some neovascularization was found from the auto-population of the body’s circulating system. The attached ECs, elastic fibers, and smooth muscle layers of the vessels were observed post-transplantation (Zhang et al., 2015).

While utilizing the biological bioreactor with the inherent flow, innate microenvironment, and progenitor cells for endothelialization can be a proper substitute for artificial bioreactor application, some bottlenecks hamper achieving desired outcomes. The first limitation is the host immune response and the possibility of graft rejection and failure (Stahl et al., 2018). A growing body of efforts has been made to understand the risk of tissue rejection in bioscaffold implantations (Xu et al., 2008). The immune response against acellular scaffolds seems to arise from three essential causalities. The first and most important influential factor in the immunogenicity of acellular structures is the presence of cellular debris and immunogenic remnants in the acellular structures (Stahl et al., 2018). Since the identification of carbohydrate and protein antigens, such as the major tissue histocompatibility complex (MHCs) at the cellular surface of the target tissue or organ, is the main reason for induction of the host immune response, it is expected that cell removal and implanting cell-free scaffolds would stimulate the immune system less (Stahl et al., 2018). Nevertheless, lack of proper decellularization and incomplete removals of immunogenic agents, such as cellular debris, immunogenic molecules, and immunogens of ECM, lead to host immune response and graft rejection.

However, the presence of remained xenogeneic antigens in the acellular structure is a highly controversial subject. It has been demonstrated that the presence of xenogeneic antigens in the decellularized structure can provoke a host immune response which is destroying the scaffold and rejecting the implanted graft (Wong et al., 2016; Eisenson et al., 2022). Moreover, the production of antibodies binding to the xenoantigens masks the ECM cues substantial for cell attachment (Galili, 2015). Conversely, the presence of xenogeneic antigens has been reported to induce complementary cell differentiation after recellularization. Cells implanted in acellular ECMs *in vivo* may require further differentiation or maturation through interactions with other xenogeneic antigens, immune or neighboring cells (Petrus-Reurer et al., 2021). Several studies have demonstrated the differentiation of embryonic stem cells and MSCs into pancreatic and neural lineages after exposure to foreign antigens (Cabrera et al., 2006; Fan and Winanto, 2020). For instance, mature stem cells derived from the human parotid gland, pancreas, and skin are differentiated into neurons by co-cultivation with xenogeneic mouse brain samples (Petschnik et al., 2011), or dopaminergic neurons that need to be further matured in the midbrain after transplantation (Fan and Winanto, 2020). As a result, it appears that the remaining xenogeneic antigens can be effective in inducing further cell differentiation and making ECs more mature. However, due to the lack of knowledge regarding antigens present in the ECM, the effect of decellularization on them, the types of cells likely to differentiate into ECs, and the quantification of immunosuppressive treatments, further research is required to elucidate these aspects more fully.

The second affecting factor is the implantation site, which is selected based on different criteria. Omentum, with a rich blood supply and subcutaneous position with low oxygen tensions, are the two chosen sites for implants (Liao et al., 2013; Khorramirouz et al., 2017). Banerjee D et al. implanted the acellular and cellular aorta scaffolds in the omentum and subcutaneous position. It was observed that in contrast to omental implants, the subcutaneous site implantation minimized subsequent immune responses (Banerjee et al., 2022). The change of ECM microstructure during the decellularization process is the third factor. The more preserved and intact it is, the less likely it induces an immune response (Wong et al., 2016). It is observed that as long as the major components of the ECM, such as collagen and proteoglycans, are maintained, a small immune response is induced, and higher efficacy is provided for the acellular scaffold (Xu et al., 2008).

As a double-edged sword, inflammation caused by immune response plays ambivalent roles in the endothelialization and cellularization process (Hortensius and Harley, 2016; Vasconcelos et al., 2019). While the immune system’s reaction to implanted scaffolds may pose a hurdle in the tissue engineering process, it is also important to note that inflammatory processes play a crucial role in the regeneration and cellularization of acellular structures (Karin and Clevers, 2016; Dziki et al., 2017). It is why immune modulation, not suppression, is intended for post-implantation of the acellular scaffold in the target host. The ECM has immune-modulating properties, including regulating critical cells such as macrophages involved in the host immune response (Dziki et al., 2016; Dziki et al., 2017; Hong et al., 2020). Macrophages and their interactions have significant impacts on the regeneration process, especially vascular regeneration (Jetten et al., 2014; Hong and Tian, 2020). Studies have shown that M1 macrophages participate in inflammation and the onset of angiogenesis, while M2 macrophages are mainly involved in vascular maturation and tissue regeneration (Hong and Tian, 2020; Martin and Gurevich, 2021). Therefore, one of the valuable methods for modulating the immune response and inducing angiogenesis in the acellular structure is macrophage phenotype polarization (Brown et al., 2012). Recently, it was observed that using vascular endothelial growth factor (VEGF) in the acellular porcine SIS structure led to infiltrated macrophage polarization to M2 phenotype, resulting in a more successful and complete endothelialization (Smith et al., 2019).

In this regard, discovering and creating biomaterials and techniques that can regulate the immune system to promote tissue repair and regeneration is the foremost challenge One viable solution is crosslinking the ECM’s proteins and structures (Sun et al., 2016). Assuming that free amino, carboxyl, and hydroxyl groups on collagen and other ECM components may have antigenic effects, crosslinking acts by covering these free groups with chemical, natural, and enzymatic agents such as glutaraldehyde (GA), genipine, or carbadiimides (Chang et al., 2002; Sun et al., 2016). Cross-linking is often used to reduce the acellular structure’s immunogenicity; however, it can be a mediator of inflammation. For instance, while GA crosslinking is effective in reducing hyperacute and acute rejection in xenogeneic tissues, it has a range of adverse effects, including immunogenicity, cytotoxicity, and calcification (Krishnakumar et al., 2019; Bhattacharjee and Ahearne, 2021; Guo et al., 2021). Furthermore, it can lead to a high crosslinking density, which may interfere with tissue regeneration and lead to chronic inflammation and foreign body response (Delgado et al., 2015; Grebenik et al., 2020). Genipin has been found to be more successful than other biomaterials at preventing scaffold rejection, reducing the inflammatory process, and enhancing the tissue regeneration rate (Chang et al., 2002; Xi-xun et al., 2010). Preclinical studies on porcine decellularized esophagus and liver demonstrated that genipin-treated scaffolds induced a shift in infiltrating macrophages towards the M2 subtype, leading to more efficient tissue regeneration (Koch et al., 2012; Wang et al., 2016). Additionally, studies have shown that genipin-fixed vascular surfaces led to relatively complete endothelialization and that cultured HUVECs attached to acellular structures were functional, resulting in an increase in cellular markers (Xi-xun et al., 2010).

More than immunogenicity, there are other limitations in biological bioreactor application, such as thrombosis formation, which occludes the implanted vascular network after implantation, and the requirement of microsurgical skills to implant the scaffold into the recipient’s body (Zhang et al., 2015). Therefore, this matter must be studied much more to regenerate viable and completely functional implanted organs *in vivo*.

## 4 The role of surface modification in endothelialization enhancement

It has been demonstrated that the attachment and survival of ECs depend on the cell-ECM interaction (Sarig et al., 2018). The remarkable architecture of scaffolds and their components with good biochemical activity and biocompatibility play a vital role in the recellularization process (Oberwallner et al., 2015). ECM proteins, including fibronectin and collagen, and the integrins mediate cell adhesion and survival (Sottile, 2004; Sgarioto et al., 2012). Recent studies have indicated that the concentration and pattern of proteins present in the ECM lead to a range of cellular responses, from apoptosis to growth and differentiation (Sottile, 2004). In addition to cell-ECM interactions, GFs are required for cell adhesion, growth, and migration (Huang et al., 2007).

**FIGURE 3:**
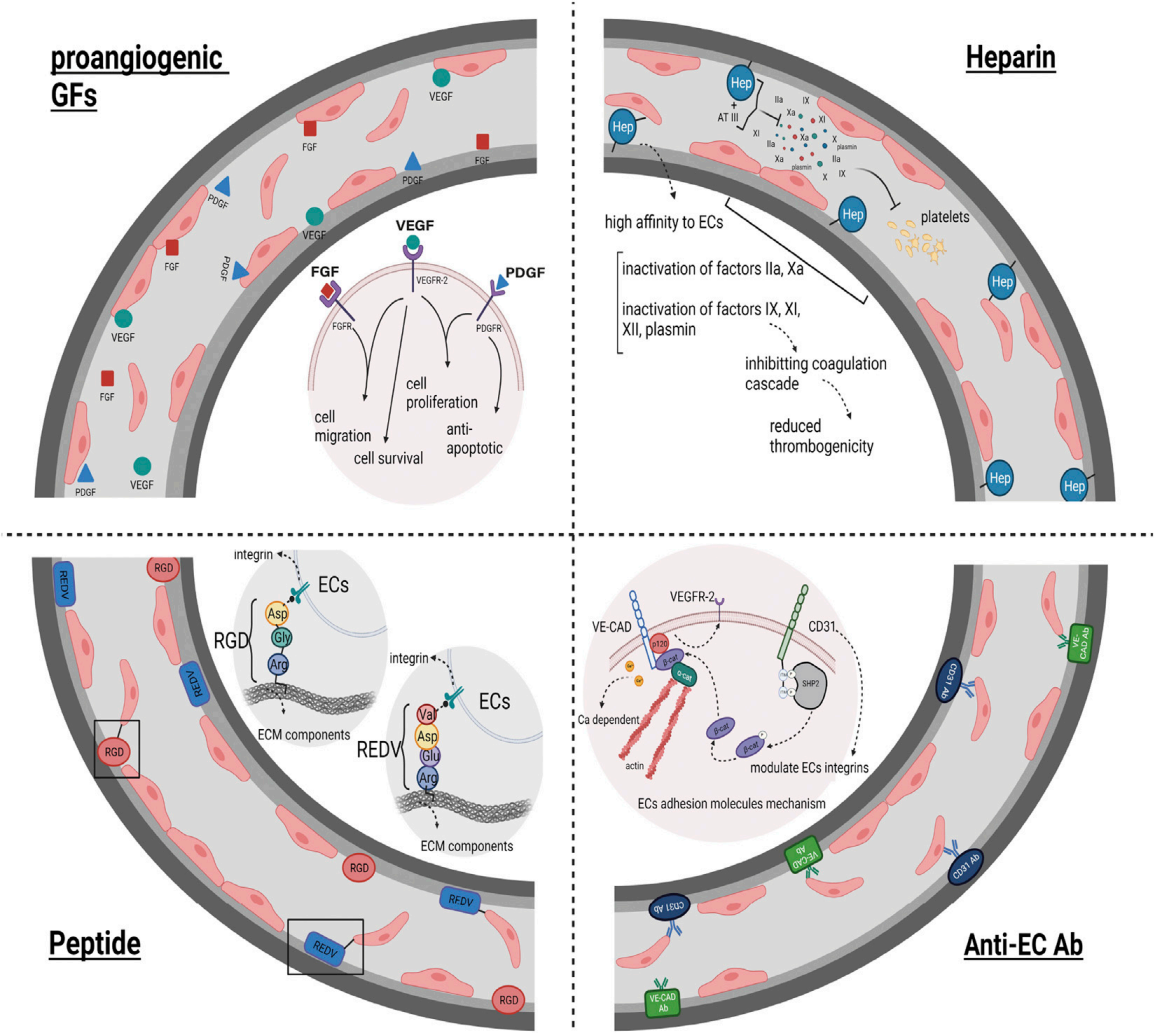
Surface Modification impact on Re-endothelialization: This figure illustrates the effectiveness of acellular structures surface modification on the result of endothelialization and ECs coverage of acellular vessels. Heparin biofunctionalization, as one of the ways to modify the acellular surfaces due to its high affinity to ECs and to disrupt the coagulation cascade and prevent clot formation in the vessels, leads to the endothelialization of the acellular vascular network. Anti-ECs Abs, as well as peptides possessing a recognition site for ECs, bind to their receptors present on ECs, subsequently increasing the cell attachment to the vascular scaffold and improving the endothelialization outcome. The utilization of proangiogenic GFs as another factor utilized in the field of surface modification of acellular structures leads to an increase in re-endothelialization and much more complete vascular ECs coverage by stimulating and increasing cell migration, cell proliferation, cell survival, and anti-apoptotic features.

In this regard, it has been displayed that a considerable percentage of binding/adhesion molecules, GAGs, and GFs remain in the acellular scaffold post-decellularization (Brown and Badylak, 2016). For instance, it was displayed that the distribution of specific ECM proteins was similar to that in native rat kidney tissue. Additionally, it was revealed that levels of the cytokines hepatocyte growth factor (HGF), Transforming growth factor beta (TGF-β), and VEGF in the acellular kidney scaffold were similar to that of the intact kidney (Guan et al., 2015). Similarly, another study investigating whole liver regeneration found that the levels of collagen, elastin and GAGs in the decellularized tissue did not significantly differ from that in the native tissue (Baptista et al., 2011). Although this appeared to lead to favorable endothelialization in vascular structures, severe damage to the three-dimensional structure of molecules and tissues caused by the strong chemicals used in the decellularization process hindered this (Poornejad et al., 2016). Thus, modifying the surface of natural materials in tissue engineering seems necessary to overcome this challenge. Surface modification is performed to improve cell activity at the material’s surface without changing the tissue’s overall skeletal integrity (Hsia et al., 2019; Li et al., 2022b; Dias et al., 2022). It can enhance re-endothelialization and prevent clot formation within the acellular vasculature. Surface modification or interface biofunctionalization in acellular structures has been done using different adhesive molecules and GFs (Zhou et al., 2019). (Which are briefly shown in Figure 3.)

### 4.1 Adhesive molecules and efficient endothelialization

Many adhesive molecules such as bioactive proteins, bioactive peptides, heparin, hyaluronic acid (HA), fucoidan, chitosan (Lv et al., 2020), antibodies (Abs), statins, and sphingosine 1-phosphate (Sun et al., 2010; Hsia et al., 2019) have been used to modify decellularized surfaces for promoting endothelialization outcomes. These molecules can be categorized into proteins, peptides, saccharides, and antibodies.

According to the bioinduction characteristics of decellularized materials and their regenerative effects, including the role of ECM on stem cell niches, angiogenesis, and tissue-specific differentiation, ECM components were used in the direction of surface modification. In this regard, fibronectin, have been utilized to coat the surfaces of engineered vascular grafts to promote autologous recellularization and graft function by facilitating the adhesion of ECs to ECM (Aubin et al., 2016). Fibronectin serves as a general cell adhesion molecule that can promote endothelial progenitor cells (EPCs) settlement and proliferation (Wang et al., 2012a). For instance, fibronectin fused into the acellular scaffold before cell seeding improved re-endothelialization. In addition, fibronectin has been used as an adhesive factor for HGF to immobilize it to the acellular scaffold (Guo et al., 2018). So that, fusing the fibronectin-HGF compound to the acellular heart valve resulted in better ECs attachment and, ultimately, much more complete re-endothelialization (Ota et al., 2005). Further, peptides, as a short chain of amino acids and building blocks of specific proteins, have been utilized as substitutes for protein due to the higher molecular protein size and their limitations in acellular vasculature modification. Among the peptides, matricryptins or matrikines can be mentioned (Bellon et al., 2004; Ricard-Blum and Salza, 2014; Ricard-Blum and Vallet, 2016). Matricryptins or matrikines are biologically active fragments released from ECM proteins such as fibronectin and collagen by proteases (Bellon et al., 2004; Ricard-Blum, 2011). It has been demonstrated that matricryptins or matrikines affect the cell and its behaviors through several mechanisms, including integrins and cell signaling pathways (Bellon et al., 2004; Ricard-Blum and Vallet, 2016). So that these bioactive peptides are involved in various biological processes, including angiogenesis, cell adhesion, chemotaxis, cell growth, migration, and differentiation (Bellon et al., 2004; Ricard-Blum and Vallet, 2016). Therefore, it has become a suitable option for modifying the surfaces of acellular scaffolds to achieve better ECs coverage along the vasculature (Lee et al., 2014). These binding sites increase cell attachment, leading to enhanced endothelialization. Peptides have a more straightforward structure, higher bioactivity, and better stability than proteins (Wang et al., 2018). RGD, REDV, GRGDSP, IKVAV, and DGEA are peptides that have been used to modify acellular vascular surfaces and improve hemocompatibility (Wan et al., 2020). In this regard, Arginyl-glycyl-aspartic acid (RGD) was utilized to modify the decellularized porcine aortic valve. HUVECs were perfused through the modified valve, and the outcome represented more remarkable cell attachment, proliferation, migration, and overall much better endothelialization (Zhou et al., 2015).

Another utilized peptide is the Arginine-Glutamate-Aspartate-Valine sequence named REDV, known as the primary recognition motif for integrin α4β1 presented on ECs (Wang et al., 2018). Devalliere J et al. reported increased EC adherence, migration, and proliferation within rat liver’s acellular vascular network and reduced platelet adhesion and aggregation using REDV-ELP (REDV fused to elastin-like peptide) conjugated scaffolds (Devalliere et al., 2018). As another peptide, Wan J et al. enhanced the re-endothelialization of rat decellularized pancreatic scaffolds and represented more outstanding survival support and proliferation of HUVECs by conjugating the GRGDSPC peptide to the acellular pancreas scaffold and subsequently seeding HUVECs *via* portal vein perfusion (Mahara et al., 2015). In another luminal surface modification, the decellularized graft was modified using a chimeric peptide of REDV conjugated with a repetitive Pro-Hyp-Gly (POG) sequence (POG7- G3REDV). This compound binds to present collagen in the ECM *via* POG sequences and facilitates the adherence of ECs through REDV. The results showed 86% of ECs were attached in POG7G3-REDV modified scaffolds, while this percentage was reported much lesser in non-modified or random peptide modified scaffolds. Additionally, after transplantation into the minipig abdominal aorta, a 4-week study of these scaffolds showed much higher vascular patency than the others (Mahara et al., 2019). As one more peptide modification study, Zhou J et al. modified PEG cross-linked acellular valves by conjugating Arg, Glycine, and Aspartate to VEGF protein. They reported that the new chimeric utilization improved the adhesion, migration, proliferation, and functionality of EPCs (Zhou et al., 2016b).

Another group of bioactive molecules used in surface modification is saccharides such as Glycosaminoglycans (GAGs). Heparin immobilization, as one of the most studied GAGs and an anticoagulant, is utilized to modify an acellular surface scaffold to increase endothelial hemocompatibility and biocompatibility (Wu et al., 2016; Xie et al., 2021). Besides the anticoagulant effects, it has been demonstrated that heparin and heparin-like polymers meaningfully improve angiogenesis and reduce the exogenous growth factor requirement due to heparin’s high affinity to ECs (Wu et al., 2016). Such that acellular grafts conjugated with heparin reduced the thrombogenicity by inhibiting platelet activation and aggregation (Mahara et al., 2020).

Considering the positive impacts of other materials and the synergy of molecules on each other, producing chimeric molecules is a more practical method to modify the acellular surfaces. Particularly, heparin has been conjugated with other proteins to generate functional molecules to increase ECs attachment. Jiang B et al. linked a collagen-binding peptide to heparin which led to more and more extended attachment of ECs to the acellular vascular bed and reduced platelets activation (Jiang et al., 2016). Heparin-gelatin, another heparin-protein compound, has been used to coat the decellularized liver based on heparin’s effects on angiogenesis. It has been found that gelatin-coated surfaces can promote cell adhesion without affecting regular migration. Evidence has demonstrated that heparin-gelatin mixtures promoted the migration and attachment of ECs to vessels. Besides, the decellularized scaffolds coated with heparin-gelatin promoted the vessel antithrombotic effect, coverage, proliferation, and functionality (Hussein et al., 2016). Heparin-VEGF, another chimeric molecule, can establish and improve angiogenesis much more than the native decellularized scaffolds. Enhanced HUVECs proliferation in the heparin-VEGF modified matrix originates from heparin immobilization and evokes from the sustained release of VEGF. A heparin-VEGF multilayer acts as an anti-thrombogenic agent, which can improve cell attachment, immigration, and proliferation (Ye et al., 2013).

One of the more feasible methods to modify an acellular scaffold is polyelectrolyte multilayer film utilization (Ye et al., 2013). Intermittent adsorption of oppositely charged polyelectrolytes at the material level is the main basis for modification in this method (Ye et al., 2012). For instance, heparin-chitosan multilayer film as a saccharide-saccharide chimeric molecule was used to coat the decellularized aortic heart valve. Significant reduction in platelet adhesion and activation, a meaningful reduction in leukocyte attachment, erythrocyte hemolysis, and blood clotting time, as well as forming a confluent monolayer of EPCs after seeding on the modified acellular valve were the results of heparin-chitosan utilization (Ye et al., 2012). Due to the antithrombotic, anticoagulant, and anti-inflammatory properties of fucoidan, a natural sulfated polysaccharide, and its high affinity to VEGF, fucoidan-VEGF polyelectrolyte multilayer film-coated porcine heart valves scaffold was used to modify the valvular structure and promote the valvular bioprosthesis. This fucoidan-VEGF is a suitable stimulus for induction and progression of re-endothelialization and, subsequently, tissue regeneration due to antithrombotic features, non-calcifying property, enhanced HUVECs attachment to the acellular scaffolds, and continuous release of VEGF in the acellular structures (Marinval et al., 2018).

HA is the other saccharide that has been used for surface modification purposes. It has been shown that different aspects of cell behavior uniquely depend on the size of surrounding HA fragments. It seems that ECs respond to HA oligomers (<10 kDa) more than long-chain HA components (1000 kDa) (Ibrahim and Ramamurthi, 2008). Although it has previously been thought that ECs react poorly to intermediate HA fragments, both oligomeric and high molecular weight HA have beneficial effects on re-endothelialization in different ways. It has been shown that large HA-1500 polymer filaments reduced the production of cumulative capillaries but increased the length of each tube due to the restriction of EC migration pathways and the prevention of capillary branching. However, small fragments of HA stimulate capillary production and have less effect on cell migration. Besides, oligomeric HA increases the expression of ECs activation markers and inflammatory cytokines more than HA-1500, leading to better endothelialization and angiogenesis (Ibrahim and Ramamurthi, 2008).

As the third category of surface modifiers, antibodies (Abs) are conjugated to the components of ECM present in the acellular structure, including collagen. On the other hand, they bind to ECs that promote cell-ECM adhesion as well as strengthen their attachment to the vascular bed due to having a particular identification site for ECs proteins (Ko et al., 2014; West-Livingston et al., 2020). The binding of anti-endothelial cell Abs to decellularized scaffolds improves the coverage of endothelial vessel wall, leads to the generation of a homogenous endothelial layer through the vascular network, and prevention of platelets activation and aggregation (Ko et al., 2014; Ko et al., 2015). Studies have used different types of anti-endothelial cell Abs, such as anti-CD31, anti-vascular endothelial cadherin (VE-CAD), anti-VEGF receptor 2 (VEGFR2), and anti-von Willebrand factor (vWF) Abs. In the meantime, anti-CD31 Ab has been introduced as the most widely used Ab in these studies (Jackson et al., 2014; Ko et al., 2014). For instance, Huling J et al. demonstrated that binding anti-CD31 Ab to the decellularized scaffold could promote EC adhesion under the physiological flow rate. Even during increasing flow rate, the ability of cell adhesion remained higher in the anti-CD31 Ab conjugated scaffold. Their study showed that about 97% of ECs were attached to conjugated scaffolds, while this percentage was half of that in deconjugated scaffolds (Huling et al., 2018). It also has been observed that the anti-CD31 Ab conjugated vascular network within the acellular porcine kidney was patent post-grafting, unlike unconjugated vessels, which were utterly blocked after implantation (Ko et al., 2014).

### 4.2 Proangiogenic growth factors and efficient endothelialization

Another promising surface modification approach to improve the integration of transplanted materials and promote cell-scaffold collaboration is biofunctionalization with GF-related molecules, especially proangiogenic GFs (Aubin et al., 2016). Angiogenesis is a multi-stage and multi-factorial process entirely regulated by a dynamic balance between proangiogenic and anti-angiogenic factors (Gerwins et al., 2000). The crucial role of proangiogenic GFs such as VEGF-A, fibroblast growth factor (FGF), HGF, platelet-derived growth factor (PDGF), and integrins and their receptors in angiogenesis is demonstrated by altering the gene expression in the target cells. Angiogenic agents bind to receptor-dependent tyrosine kinase that induces the enzymatic activity of these receptors. This chain of changes eventually leads to cellular activities such as migration, proliferation, and differentiation (Gerwins et al., 2000; Lohela and Alitalo, 2003; Herzog et al., 2011). Angiogenic GFs such as VEGF and FGF force cells to produce many endothelial proteinases, matrix metalloproteinases, and plasminogen activators that degrade vascular BMs, which allow cells to penetrate the adjacent matrix. As positive angiogenic factors, integrins (such as avb3 and avb5) enable cells to migrate in the degraded matrix to form a new vessel (Roberts and Palade, 1997; Hellström et al., 2001). Therefore, considering the prominent role of proangiogenic GFs in organ regeneration, utilization of endogenous and exogenous GFs are the two approaches proposed to improve endothelialization.

Studies have shown that several molecules involved in inflammatory and angiogenic cascades are preserved in the acellular ECM after decellularization and the ECM acts as a reservoir for GFs (Peloso et al., 2015; Hussein et al., 2016). In addition, the concentration of proangiogenic GFs within the acellular scaffold is essential for re-endothelialization. It has been reported that organ-specific functions and the generation of a tissue-like structure are enhanced by a high concentration of endogenous GF and the co-addition of various types of GFs (Nakamura and Ijima, 2013). Although GFs are retained in the acellular structure following decellularization, their concentration decreases (Hussein et al., 2016). It has been observed that only 52% and 30% of VEGF and basic fibroblast growth factor (bFGF) were maintained in decellularized vessel scaffolds, respectively, which indicates that modification is required to achieve the desired results (Hussein et al., 2016).

Exogenous GFs have been utilized in various ways to promote endothelialization. In the most basic method, several studies used GFs in solution by adding them to the cell culture medium or perfusion culture (Shirakigawa et al., 2012; Zhou et al., 2016a; Devalliere et al., 2018). Although this approach led to improved adhesion, proliferation, differentiation, and migration of ECs, the short half-life of GFs and the requirement for their long-term effectiveness has been their limiting factor (Iijima et al., 2018; Ren et al., 2019). Therefore, other approaches, such as the induction and secretion of GFs by perfused suitable cells and the immobilization of GFs on the acellular structure, were welcomed and employed.

In the first approach, studies have demonstrated that aside from the proangiogenic GFs preserved in the acellular scaffold, the stem cells used in recellularization secrete GFs and cytokines, which are helpful in re-endothelialization. VEGF and bFGF are secreted from MSCs over paracrine mechanisms, which can enhance ECs function (Xu et al., 2014). ECs can also stimulate vasculogenesis and angiogenesis by secreting GFs such as VEGF and bFGF (Wang et al., 2012b). Further, cell preconditioning can increase the level of expression and secretion of GFs by using various factors such as flow, pressure, stress, oxygen, and nutrient supply gradients (Buchanan et al., 2014). It has been demonstrated that preconditioning with oscillating pressure, flow, and mechanical strain increases the GFs expression and promotes angiogenesis and endothelialization of the acellular tissue-engineered blood vessels (Yazdani et al., 2009). As the second approach of exogenous GFs delivery, studies have been conducted to investigate the immobilization of GFs on the ECM substrate, improving their long-term stability by controlling GFs’ release and concentration within the scaffold (Liu et al., 2022). Immobilized VEGF on the acellular luminal surfaces absorbs and captures circulating blood monocytes with high specificity that differentiates into ECs (Iijima et al., 2018). The result of immobilized VEGF was complete acellular vascular graft endothelialization after 1 month with proper patency and the formation of an efficient and functional media layer after 3 months (Smith et al., 2020). Another study conducted a vascular graft functionalization with heparin-VEGF to implant it into the ovine animal model to evaluate endothelialization and inflammatory responses. Acceptable patency and fully endothelialized lumen indicated the well bio-functionality of VEGF 1-month post-implantation. Besides, VEGF may capture macrophages from the bloodstream and create an anti-inflammatory microenvironment by polarizing the infiltrated macrophages to the M2 (anti-inflammatory) macrophage phenotype, which will promote tissue regeneration (Smith et al., 2019). bFGF, as another GF, has been utilized to modify the acellular bovine pericardial patches. Evidence demonstrated a desirable endothelialization on the endocardial (inner) surface of patches after implantation to the rat ventricles (Chang et al., 2007). Furthermore, GFs’ synergistic effect should also be considered and employed. The synergy of GFs such as FGF and VEGF can motivate the angiogenesis and tubular formation of ECs (Gerwins et al., 2000).

A prominent approach to preserve proangiogenic GFs concentration in a specified and practical range is to control GF release. One of the used methods is encapsulation in the microspheres (Aikawa et al., 2012; Li et al., 2017; Zhou et al., 2019). Encapsulation of GFs is done to achieve a much-controlled level of GFs within the scaffold and promote re-endothelialization in acellular scaffolds by covalently modifying the biomaterial with cytokines. Additionally, encapsulating cells beside GFs decreases the possibility of cell aggregation and makes it possible to seed more cells. In this respect, Zhou J et al. used a nano-drug-controlled release system to produce a hybrid valve (Zhou et al., 2019). They encapsulated VEGF into polycaprolactone with a cell seeding approach and observed an enhancement in the endothelialization of tissue-engineered heart valve scaffold (Zhou et al., 2019). In another study, researchers utilized semipermeable microcapsules containing genetically modified fibroblasts to secrete VEGF in the implanted xenogeneic acellular dermis to improve angiogenesis and vascularization in the wound. Their comparison between contained microencapsulated cells’ composite skin and the control group demonstrated that angiogenesis, cell survival, and the healing rate were much better in the microcapsule-containing group (Han et al., 2010).

Taken together, proangiogenic GFs have significant effects on re-endothelialization and supporting angiogenesis. Many methods have used GFs to provide a basis for the endothelialization of acellular vasculature; future experiments are still needed to determine their exact effectiveness.

## 5 The role of cell type on endothelialization

**FIGURE 4:**
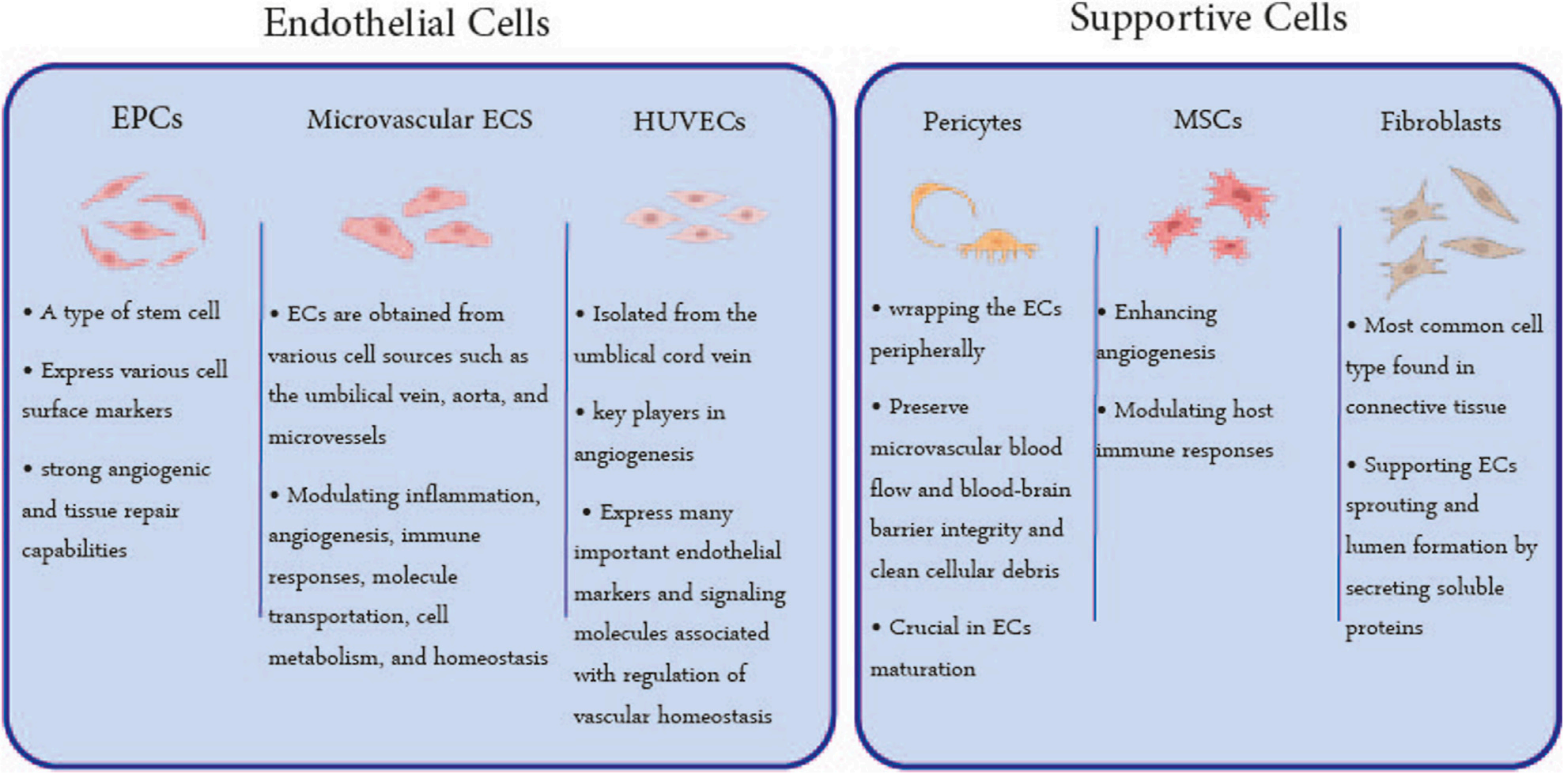
Utilized Cell Types in Re-endothelialization: The cells utilized in the endothelialization of acellular scaffolds can be classified into two general categories: ECs and supporting cells. EPCs, microvascular ECs, and HUVECs are the most used ECs, and MSCs, pericytes, and fibroblasts are supporting cells whose characteristics are mentioned in this figure.

Active vascular remodeling, an action that imitates lumen formation during vascular development, is needed to generate a perfusable vascular lumen from decellularized vessels (Ota et al., 2005; Scarritt et al., 2018). Considering the different capabilities of the cell types, selecting appropriate cells could influence the re-cellularization process (Lin et al., 2019). Therefore, cell type is the fourth and final issue in acellular structure re-endothelialization (Ciampi et al., 2019). The vascular structure consists of vessels with different sizes, anatomy, physiology, and cellular arrangements. Capillaries, as the smallest member of the network, are composed only of ECs, while there are two series of endothelial and supporting cells in the wall of larger vessels. ECs form the inner lining of blood vessels while supporting cells to establish vascular function and structures by releasing cytokines and growth factors and tightening cell adhesions. In this regard, the cells used in re-endothelialization studies can be grouped into two general categories of ECs and supporting cells (Doi et al., 2017; Xu et al., 2019) (Figure 4).

### 5.1 Endothelial cells for vessel recellularization

In intact tissues, ECs play a critical role in re-endothelialization, modulating inflammation, angiogenesis, immune responses, molecule transportation, cell metabolism, and homeostasis by expressing different genes and activating a variety of signaling pathways (Weymann et al., 2013). They can even stimulate angiogenesis by secreting GFs (Wang et al., 2012b). Utilized ECs are obtained from various cell sources such as an umbilical vein, aorta, and microvessels. In order to perform an endothelial coverage on the vascular network in engineered tissue, decellularized rat iliac arteries have been re-endothelialized by seeding skin microvascular ECs onto the luminal surface of the acellular matrix. *In vivo* implanted arteries showed partial ECs coverage with platelets adhered to the bare cites at 1 month and a similar structure to native vessels with no detected thrombosis 3 months post-transplantation (Dall’Olmo et al., 2014). In another study, MS1 pancreatic ECs were infused through the acellular kidney scaffolds using a combined static and ramping perfusion seeding approach. It was shown that cell-seeded scaffolds significantly inhibited platelet adhesion on cell-attached surfaces compared to the substantial platelet deposition found on the unseeded scaffold (Ko et al., 2014).

Meanwhile, it has been demonstrated that the apparent differences in gene expression, function, and the profile of adhesion molecules distinguish the large-vessel ECs from the microvascular ECs (Ribatti et al., 2002). It has been revealed that arterial ECs in endothelialization along the vascular walls are often discovered in large blood vessels (50–75 μm diameter) and rarely in capillaries. The venous ECs mainly cover the walls of small and medium blood vessels (25–50 μm diameter), and the microvascular ECs are heavily attached throughout the vascular tree, including capillaries (Scarritt et al., 2018). In an experimental investigation, decellularized rat lung scaffolds were re-endothelialized by co-seeding with rat pulmonary artery endothelial cells (PAECs), rat pulmonary vein endothelial cells (PVECs), and rat pulmonary microvascular endothelial cells (MVECs) (Scarritt et al., 2018). PAECs were infused in the pulmonary artery. PVECs were seeded into the pulmonary veins, and MVECs were dual-seeded to cover both sides of the vascular microstructure. The results showed that although the amount of apoptosis in PVECs was higher than PAECs and MVECs, all rat-specific ECs had a higher level of proliferation and lower levels of apoptosis than HUVECs (Scarritt et al., 2018). Furthermore, the combined infusion of arterial, venous, and microvascular ECs, instead of using a single endothelial cell type, resulted in tight junctions between ECs, forming a mature endothelium covering the decellularized vasculature. Therefore, using organ-specific ECs in addition to a combination of arterial, venous, and microvascular ECs can be influential in attaining complete endothelialization in the acellular scaffolds (Scarritt et al., 2018).

EPCs are another type of cell used in the re-endothelialization of decellular blood vessels and heart valves (Zhou et al., 2016a). By expressing specific cell surface markers, these circulating cells stick to endothelium at the destination site and cooperate in new vessel development (Yoder, 2012). EPCs have higher angiogenic potential than ECs *in vivo* (Wang et al., 2012b). To reconstruct the vascular network, some studies have been done to recellularize the acellular pancreatic scaffold with EPCs. For instance, the vasculature of the acellular scaffolds has been successfully endothelialized by EPCs from rat bone marrow (Zhou et al., 2016a). Also, it has been revealed that EPCs derived from bone marrow are precisely attached to decellularized vessels and generate new blood vessels on the acellular pancreatic scaffold (Guo et al., 2018).

HUVECs, primary cells isolated from the human umbilical vein, are another EC subtype used to cover the acellular vascular tree. In this respect, following circulation culture, HUVECs were infused through the portal vein (as the central duct of the liver). The study achieved an endothelialized vascular network without any observed leakage in decellularized livers (Shirakigawa et al., 2012). Further, the human heart valve (Weymann et al., 2013) and decellularized rat lung (Scarritt et al., 2018) are the other studied tissues that benefited from HUVECs utilization in the re-endothelialization. Their outcome represented proper cell attachment, migration, and complete coverage of endothelial cells on the human heart valve and the acellular lung vasculature.

### 5.2 Supporting cells and Co-Culture for vessel recellularization

Supporting cells, such as MSCs, pericytes, and perivascular cells, play a variety of roles, including improving ECs viability and proliferation and inducing angiogenesis by producing various GFs and ECM components as well as stabilizing newly formed vessels (Shafiee et al., 2021). Therefore, the co-cultivation of supporting cells with target tissue cells should be a practical approach to induce angiogenesis and vascularization within the engineered constructs (Wang et al., 2012b).

MSCs, as one of the supporting cells, have been utilized in acellular scaffold endothelialization due to their complementary role in enhancing angiogenesis and modulating host immune responses (Ahmed et al., 2021). It has been shown that GFs, such as VEGF and FGF, could be secreted from bone marrow MSCs through paracrine mechanisms in the acellular ECM. It has been suggested that MSC-derived ECMs can mimic vascular ECM microenvironments and support HUVECs growth (Xu et al., 2014). In this direction, perfusing co-culture of HUVECs and human MSCs in the acellular porcine cardiac ventricular ECM led to the endothelialization of the structure and formation of a vascular network (Sarig et al., 2015). Wang Z et al. also co-cultured ECs with smooth muscle cells (SMCs) in decellularized small intestinal submucosa scaffolds. It was a promising method for enhancing tissue vascularization and neo-capillary formation. This study reconfirmed that co-cultivating ECs with target tissue cells can induce functional vascularization in engineered tissues (Wang et al., 2012b). Another co-cultured cell study represented improvements in the re-endothelialization of lung scaffolds and achieved 75% endothelial coverage by co-seeding HUVECs and human MSCs (Ren et al., 2015). Further, ASCs were co-cultured with ECs to re-endothelialize the acellular rat pulmonary vasculature. The results showed that ASCs differentiated into pericytes and subsequently led to improved endothelialization and much more stabilized endothelial cells and newly formed vessels (Doi et al., 2017).

Pericytes, as multi-functional cells in microcirculation, exist peripherally in the capillaries and venules around the ECs (Scarritt et al., 2018). They preserve microvascular blood flow and blood-brain barrier integrity and clean cellular debris. Moreover, according to their multi-potential features, they can differentiate into smooth muscle cells or other mesenchymal cells during *in vitro* differentiation and *in vivo* vascular remodeling. Pericytes can also communicate with the ECs *via* direct contact and the paracrine signaling pathway. Moreover, they are associated with EC differentiation and proliferation, which play a crucial role in EC maturation, and lead to a better angiogenesis process. In this regard, umbilical cord pericytes have been utilized to functionalize the conduit made of ECM. Observations indicated the pericytes’ ability to enhance HUVECs migration, ECM remodeling, and vessel formation (Cathery et al., 2020).

Fibroblasts, the most common cell type found in connective tissue, participate in angiogenesis. Dew L et al. improved the endothelialization of the decellularized intestine scaffold by continuous perfusion and infusing human dermal fibroblast co-cultured with human dermal microvascular endothelial cells (HDMECs). Their results showed the deposition of cells on the surface of vascular structures 1 day after graft transplantation. They represented vessels covered by HDMECs after 5 days in perfusion-based culture (Dew et al., 2016). Further, human skin fibroblasts co-cultured with HUVECs improved the endothelialization of the acellular pancreatic scaffold, and more vessels were formed in a shorter period. Finally, the endothelialization enhancement may be due to an increase in GFs secretion subsequently to HUVECs and fibroblast co-culture (Xu et al., 2019). The common cell types used in the re-endothelialization of various decellularized organs are shown in Table 1.

**TABLE 1:** The application of cell type on Re-endothelialization.

Type of cells			Organ	Animal	Ref
Application of endothelial cells (ECs)	Microvascular ECs		Decellularized arteries	porcine	Yazdani et al. (2010)
			Iliac arteries	Rat	Dall’Olmo et al. (2014)
			Lung	Rat	Le et al. (2017)
			Kidney	porcine	Ko et al. (2014)
			Lung	Rat	Stabler et al. (2016)
			Pulmonary Heart valves	Ovine	Lichtenberg et al. (2006)
			Liver	porcine	Hussein et al. (2016)
			Liver	Rat	Devalliere et al. (2018)
			Heart	Rat	Robertson et al. (2014)
	Combination seeding of rat pulmonary artery endothelial cells (PAECs) and rat pulmonary vein endothelial cells (PVECs), and rat pulmonary microvascular endothelial cells (PMVECs)		Lung	Rat	Scarritt et al. (2018)
	EPCs		Liver	Rat	Zhou et al. (2016a)
			Heart Valve	Pig	Zhou et al. (2016b)
			Aortic heart valve	Porcine	Ye et al. (2013)
	HUVECs		Decellularized ovine pulmonary heart valve cusps (dPVCs) and decellularized rat aortic grafts (dAoGs)		Aubin et al. (2016)
			Liver	Rat	Shirakigawa et al. (2012)
			Heart Valve	Human	Weymann et al. (2013)
			aortic valve	Porcine	Zhou et al. (2019)
			Liver	Porcine	Mao (2017)
			Kidney	Rat	Mei et al. (2016)
Co-cultivation of endothelial cells and supportive cells (ECs + Supportive Cells)	Mesenchymal Stem Cells (MSCs)	human mesenchymal stem cells (hMSCs) and rat adipose-derived stem cells (rASCs)	decellularized porcine myocardium slices (dPMS)		Kc et al. (2017)
		Murine Embryonic stem cell	Kidney	Rat	Ross et al. (2012)
	Fibroblasts	human dermal microvascular endothelial cells (HDMECs) and human dermal fibroblasts (HDFs)	decellularized natural vascular scaffold from rat intestine		Dew et al. (2016)
		human skin fibroblasts and human umbilical vein endothelial cells (HUVECs)	Pancreas	Rat	Xu et al. (2019)
	Smooth Muscle Cells	endothelial cells (ECs) were co-cultured with smooth muscle cells (SMCs)	decellularized small intestinal submucosa scaffolds	Porcine	Wang et al. (2012b)

## 6 Future perspective

Despite the progress made in the development of re-endothelialization approaches to create an efficient and functional endothelialized vasculature in tissue engineering, there remain certain bottlenecks that must be addressed. These barriers are mainly present in the D&R processes.

At present, decellularization methods have been successful in eliminating cellular components and DNA contents from the cellular scaffold. However, these protocols have largely ignored the effect of scaffold microstructure and its components on subsequent re-endothelialization (Dal Sasso et al., 2020). Research has demonstrated that variation in the microstructure and amount of ECM components (e.g., GAG, elastin, and fibronectin) can influence endothelialization outcomes. Therefore, further approaches should be undertaken in order to maintain ECM structure and endothelialization, such as physical methods (e.g., agitation, perfusion, and ultra-sonication), as well as chemical and enzymatic approaches (Dew et al., 2016; Khajavi et al., 2021). Additionally, inter-organ variations should be considered during decellularization, and the decellularization process should be adapted accordingly, depending on factors such as the target organ’s density, fat content, and thickness (Petersen et al., 2010; Baptista et al., 2011). This would involve adjusting the type of decellularizing agents, their concentration, solution pH, temperature, and decellularization time (Baptista et al., 2011; Daugs et al., 2017; Devalliere et al., 2018; Zhang et al., 2022).

In the realm of recellularization, artificial and biological bioreactors have provided endothelialized acellular vasculature; however, complete endothelialization has yet to be achieved. The lack of specific settings in artificial bioreactors (including flow rate, pressure, and shear stress) for each organ, as well as immunogenic challenges arising from transplanting acellular structures, are obstacles that should be fully addressed in further research.

Surface modification was also discussed as another influential issue in the endothelialization of acellular structures. Adhesive molecules and GFs have been used to improve ECM structure and endothelialization levels (Ye et al., 2013; Marinval et al., 2018). Despite the promising results, the translation of these studies to human clinical trials remains a primary concern. The molecules and structures discussed in this study are all biocompatible, and many of them, such as heparin, fucoidan, HA, and GFs, have been used in other clinical trials (Eppler et al., 2002; Hugl et al., 2009; Pulli et al., 2010; López-Ruiz et al., 2019). In addition, due to the potential xenogeneic source of some of these molecules, several of them may need to be produced recombinantly to reduce the risk of introducing unwanted xenogeneic residues into the scaffold (Eppler et al., 2002; Ferrari et al., 2014). With all interpretations, it is necessary that all molecules used meet the standards of the Food and Drug Administration (FDA) and receive approval.

Moreover, alternative approaches should be explored to improve re-endothelialization results. Exosomes, which are extracellular vesicles containing RNA, DNA, proteins, and lipids, may serve as potentially beneficial for complete endothelial coverage (Nazerian et al., 2021; Wang et al., 2021). Further research is needed to verify their effectiveness. Additionally, the ratio between ECs and supporting cells, as well as the sources of cells used and their capacity for migration and endothelial differentiation, should be optimized. Human MSCs can be isolated from adipose tissue, bone marrow, umbilical cord blood, placental tissue, and Wharton’s umbilical cord jelly (Main et al., 2021), while ECs can be derived from the umbilical vein, aorta, or small vessels (Majewska et al., 2021). Placenta-derived cells, which can be easily obtained and differentiated into ECs, may serve as a promising source, which should be investigated in future studies (Yazdanpanah et al., 2015; Abbasi-Kangevari et al., 2019). Additionally, further animal and *in vivo* studies should be conducted to evaluate the compatibility and function of re-endothelialized constructs.
